# Lessons learned from the first 50 thyroidectomies with Harmonic Focus Curved Shears – technical note

**Published:** 2014

**Authors:** R Iorgulescu, D Badanoiu, A Lupu, C Cucu, D Niculescu, N Iordache

**Affiliations:** * Emergency Clinical Hospital “Sf. Ioan” Bucharest; ** National Institute of Endocrinology “CI Parhon” Bucharest

**Keywords:** thyroidectomy, harmonic focus curved shears, hemostasis, thyroid

## Abstract

Precise and safe hemostasis is necessary for successful thyroid surgery. In this respect, the advent of the ultrasonic surgical device Harmonic Focus Curved Shears (HFCS) from Ethicon Endo-Surgery constituted a major progress in the domain by its multiple capabilities of dissection, grasping, vessel sealing and transecting.

The paper presents the initial experience of 50 cases with this device of a surgical team with special interest in endocrine surgery, mostly thyroid and parathyroid. The thyroid conditions for which surgery was indicated were: diffuse toxic goiter in 8 patients; multinodular toxic and nontoxic goiter in 30 patients; autonomous nodule in 2 patients; 2 patients with benign nodules at fine needle aspiration biopsy (FNAB); 4 patients with nodules positive for carcinoma at FNAB, among them 2 with unilateral cervical lymph nodes enlargement; 4 patients with highly suspect nodule on FNAB. The types of surgery performed were 4 hemithyroidectomies and 46 total thyroidectomies, 2 in association with unilateral functional neck dissections. We had 4 intraoperative hemorrhagic incidents, all in the first 15 cases and imputable to lack of expertise and improper usage of the device. We registered the following noticeable postoperative complications: 1 cervical hematoma from an arteriolar source in sternothyroid muscle demanding prompt reintervention; 8 hypocalcemias and 2 vocal cord paresis, none of which permanent. We remarked several advantages with HFCS: no necessity of changing instruments, fluentness of the intervention and more comfort for the operating team, reduced operating time, safe hemostasis. Some important tips and tricks with the usage of the instrument are presented.

## Introduction

Thyroid is the most vascularized organ in human body with a blood flow of approximately 8 ml/100g/s, which in thyreotoxic states can reach double values of 16 – 17 ml/100g/s [**1**]. So bloody was thyroidectomy at its beginnings that it makes the American academic trauma surgeon Samuel D. Gross (1805 – 1884) assert: “No sensible man will, on slight consideration, attempt to extirpate a goitrous thyroid gland every stroke of his knife will be followed by a torrent of blood and lucky will it be for him to finish his horrid butchery.” [**2**]

The surgical technique had not suffered major modifications from that described at the end of the XIX-th century by Theodor Kocher and Theodor Billroth and the basic instrumentarium remained more or less the same. 

The majority of non-specific and specific complications of thyroidectomy derive from precipitate and, sometimes, too impetuous (i.e. careless) maneuvers for obtaining hemostasis. The main principles of safeness and efficiency to be respected are the same through the decades:

1. Identification and ligation of the vessels

2. Identification and protection of the inferior laryngeal nerve (ILN)

3. Identification and protection of the parathyroid glands, the last two being directly dependent on the first [**3**]. So intraoperative and postoperative bleeding remains the main concern of thyroid surgeons.

Hemostasis can be obtained by several means.

Traditionally this is achieved by ties, sutures or clips, which are time-consuming, need changing of instruments and hands between surgeon and (trustworthy!) assistants, imply excessive manipulation of tissues and carry the risk of knot or clip slippage.

Monopolar diathermy passes current through the patient, a fact that can interfere with metal implants or pacemakers and can cause skin burns. More important is the almost unacceptable lateral thermal diffusion up to 15 mm [**4**]. Bipolar diathermy causes less lateral thermal damage, up to 6 mm [**5**], but its use is limited to very small vessels [**5**, **6**].

**Fig. 1:**
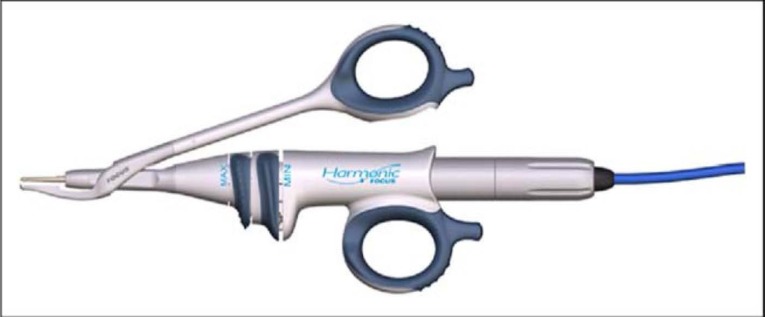
Harmonic Focus Curved Shears 9 cm. with the hand piece HPBlue

All the above-mentioned have become obsolete with the emergence of newer technologies available to thyroid surgeons and not only. Such novel hemostatic devices are:

- The electrothermal bipolar vessel sealing system (Ligasure, Valleylab, USA) with its handpiece LigasurePrecise LS1200 is extremely useful in thyroid surgery and with which the main author has an experience of more than 200 operations in the last 5 years.

- The new harmonic scalpel device for thyroid surgery Focus Curved Shears 9cm (HFCS9), which was launched in 2007, won the bronze medal in 2008 at International Design Excellence Awards (IDEAs) and caught attention and interest by its multiple capabilities of dissection, grasping, vessel sealing and transecting (**Fig. 1**).

## Patients, Materials and Methods

The aim of the study is to contribute for the younger surgeons and the beginners with the use of harmonic scalpel in thyroid and parathyroid surgery with some practical advices, some tips and tricks with the HFCS9, in order to prevent them gain an undeserved hostility towards the instrument and to fully benefit from its outstanding advantages.

Under no circumstances does this study claim to prove what have already been proven by many prospective randomized trials, i.e. the superiority of this hemostatic method when compared to others, especially in terms of operative time, in association with the same outcomes regarding postoperative hypoparathyroidism and ILN palsy.

We retrospectively reviewed the prospectively collected data of 50 patients who, during a period of 15 months (January 2013 – March 2014), underwent total thyroidectomy or hemithyroidectomy performed by the same senior consultant surgeon having an operation load of around 50 – 60 such cases per year in the last 8 years.

The demographic characteristics of the group are shown in (**Table 1**).

**Table 1:** Demographic characteristics

Demographics	Harmonic group N = 50
Age (years) medium (range)	47,8 (26 – 77)
Sex (F/M)	42/8
Thyroid volume (mean ± SD), ml (as measured by ultrasonography)	47,2 ± 14,3 (14 – 71)

The thyroid diseases for which the surgical treatment was indicated by the endocrinologists are shown in **Table 2** and **Graphic 1**. All patients had a preoperative calcemia determined and corrected for the values of plasma albumin and a preoperative vocal chords exam by indirect laryngoscopy. Those having hemorrhagic diathesis and preoperative vocal cord paresis or hypocalcemia were excluded from the study.

**Table 2:** Thyroid conditions – preoperative diagnosis

Thyroid conditions – preoperative diagnosis	Nr. patients
Diffuse toxic goiter	8
Multinodular goiter toxic and nontoxic	30
Autonomous nodule	2
FNAB + nodule for papillary or follicular carcinoma	4
FNAB highly suspect nodule	4
FNAB benign nodule	2
Total	50

**Table 3:** Type of surgery (TT – total thyroidectomy; ULMRND – Unilateral modified radical neck dissection; HT – Hemithyroidectomy)

Type of surgery	Nr. patients
TT	44
TT + ULMRND	2
HT	4
Total	50

**Graphic 1:**
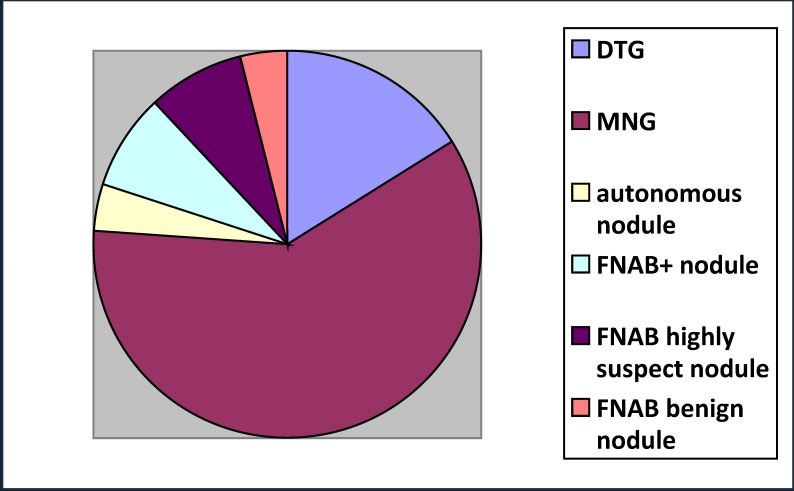
Conditions for which surgery was indicated (DTG – Diffuse toxic goiter; MNG – multinodular goiter; FNAB – fine needle aspiration biopsy)

For the 50 patients selected we have performed 4 hemithyroidectomies and 46 total thyroidectomies, among them two associated with unilateral functional neck dissection in 2 patients having FNAB firmly positive thyroid nodules with lateral cervical lymph nodes enlargement proved by ultrasonography.

The principle of the method with the harmonic scalpel is that a 110 volts generator (G11 for HFCS9), with a power scale from 1 to 5 (the higher the level means more cutting and less coagulation and vice versa; it is by default set at a minimum of 3 and a maximum of 5) send impulses of alternative current to a hand piece. The hand piece HPBlue, lighter than its predecessors and specially designed for FCS9, incorporates an ultrasonic transducer (a core of piezoelectric crystal, under pressure, between 2 metal cylinders) that vibrates at its natural harmonic frequency of 55.500 Hz [**3**], thus producing mechanical energy transmitted to the tip of the active blade in the form of excursions of 50 to 100 micrometers. The effects are heat of about 60 – 80 °C (lower than 80°C and more associated with traditional diathermy methods) and concomitant separation and coagulation without desiccation and charring [**8**].

The protagonist of the process is the Harmonic Focus Curved Shears, which is such a versatile tool, specially designed for the multiple above-mentioned tasks and which can be hold in hand like classical instruments (**Graphic 1**). Its distal part is gently curved, being thus suited for precise dissection. The lateral thermal spread at its active arm is equal to or less than 3 mm. It is indicated for cutting and coagulating vessels of less than 5 mm. 

The main author used it in all the steps of the surgical procedure, starting with division of the median raphe or the strap muscles transversally (when necessary) and ending with the ligament of Berry and detachment of the thyroid from the trachea, obviously after mandatory identification and exposure of the ILN (in its distal segment, above the intersection with the inferior thyroid artery) and, at least, the superior parathyroid glands.

## Results

We have registered four intraoperative hemorrhagic incidents (8% of the total number of cases), all of them in the first 15 cases. Two of them were from the vessels of the superior pedicle, in situations where the approach to the upper pole of the lobe was rather difficult and two of them at the severing of Berry’s ligament. They were resolved by ligatures or, respectively, by monopolar cautery, with attentive ILN protection, and could not be imputed to the method or instrument. Instead, we think that lack of experience and inadequate use were the cause, i.e. too large a vessel in one case, a precipitous activation at level 5 in one case and, presumably, too much traction in the in two cases when the source of bleeding was the ligament of Berry.

One postoperative compressive cervical hematoma necessitated return to the theatre 2.5 hours after the operation (2% incidence of early reoperation for bleeding). Its source was arteriolar in nature, from one end of the sectioned, retracted and not sutured back sternothyroid muscle.

Two patients had incisional bruises and three of them seroma that required two or three punctures and evacuations.

Eight patients had postoperative symptomatic hypocalcemia, with total calcium between 7 and 8 mg/dl (1,7 – 2 mmol/l) that responded well to supplementation with oral Calcium 2g per day and Alfa D3 1 mcg per day. None of them required calcium supplementation more than 4 months. Thus, the rate of postoperative temporary hypocalcemia (hypoparathyroidism) was 17.4% (8 from 46 total thyroidectomies). Among these eight patients, three had Graves’ disease and one had a lymph node dissection of the central compartment, both entities being recognized as risk factors for postoperative hypocalcemia [**9**, **10**].

We have recorded hoarseness in two patients and the postoperative laryngoscopic examination confirmed the right vocal cord immobility and fixation in adduction. Both sufferings were temporary, one for 6 weeks and one for 4 months. Unfortunately, we do not have the technical possibility to perform direct laryngoscopy in our unit. The rate of postoperative temporary vocal cord paresis was 4% (2 from 50 patients) or, more correctly, 2% of the nerves at risk (2 from 96 nerves at risk). Like in many other centers, perhaps the true incidence of this complication is severely underevaluated [**11**, **12**]. We always identify and expose the last 2 to 3 cm. of the ILN, above its intersection with the inferior artery, on both sides.

We left a drain for the first 24 postoperative hours; during that time, the patients were kept in the ICU for a better surveillance, should any life threatening complication appear.

The great majority of patients were discharged at 48 hours after the operation, with no difference between hemithyroidectomy and total thyroidectomy. None of them required re-admission for reasons related to surgery.

## Discussion

Bleeding is still a feared enemy of the thyroid surgeon and that may be because on its efficiency more or less directly depend the integrity of other cervical structures and so the outcome of the operation. Among the many means of obtaining hemostasis, some new technologies, techniques and devices, that came on the scene in the last 15 years, were gradually adopted by surgeons and are now preferred due to the multiple advantages they offer.

Voutilainen was the first to report a thyroidectomy with the Harmonic system (Ultracision) in 1998 [**13**] and to demonstrate an important time saving of 10 – 35% with the use of this technology [**14**], a thing that we can also confirm. Since then, many papers reporting results of trials or reviews of the literature underlined the superiority of the method and Harmonic Focus Curved Shears device stand alone in this respect.

The technology implies that no electrical energy, no current go to or through the patient, so there is no neuromuscular stimulation and, consequently, no sudden muscular contraction. Additionally there is no danger of electrical skin burns at the site of the neutral electrode or contact with other metallic parts of the table.

FCS9 is such designed like a classical surgical instrument that the surgeon’s hand is almost and already accustomed to it. It is multifunctional, being capable of carrying out the following actions: dissection (separation of tissue), grasping (prehension), traction, coagulation and cutting. It does all these with less smoke, less tissue sticking and no charring.

Reliable hemostasis is obtained with fine control and precision and, most important of all, with minimal lateral spread, of maximum 3 mm [**15**]. Nevertheless, we think that a safety limit (distance) of 5 mm from important structures that have to be protected (like ILN or parathyroid glands) is a good and judicious choice.

All the above-mentioned make the succession of operating steps very fluent and less time consuming, not to mention the comfort of the team. Talking about the team, it can be reduced to a minimum of two, as very few instruments are used and there is no need to interchange and intercross hands and instruments. More, the assistant can be a less experienced one because no ties and knots are used, so no danger of slippage does exist. All the same, there is no need of excessive muscular traction because the tip of the instrument is ideally suited for access in narrow spaces (for example the interruption of the superior vascular pedicle). Consequently, some studies demonstrated that the incision could be reduced with the use of FCS9 [**16**].

Although the producer indicates that it can be utilized for vessels up to 5 mm, our experience leads us to use it for vessels up to 3 mm, like thyroid arteries and veins are in most of the cases. In thyreotoxic states (Graves’ disease), long standing and/or big goiters and whenever we envisage difficulties in obtaining hemostasis, we think that a better choice is to use LigasurePrecise LS1200 or to fall back on classical hemostasis (vascular clips or clamp and tie). For cases that are considered in-between or when the surgeon does no feel very confident, we recommend the ”double coagulating” technique, first described by Siperstein in 2002, which implies a first sequence of coagulation without cutting proximally (level 3 for 3 seconds) and then a second sequence of coagulation and cutting distally, near the thyroid (at level 3, but longer or directly level 5) [**17**, **18**].

The technique described above and the general use of FCS9 require gradually acquisition of expertise and we appreciate that at least 10 operation is a reasonable “learning curve” before one can fully profit of the advantages it offers and at the same time stay afar from danger. By “danger”, we mean, for example, excessive traction in the moment of activation, a mistake that can lead to inadvertently cutting before a coagulum was formed, thus producing accidental bleeding. This is the kind of incident that can make the young or inexperienced surgeon to draw the unfair conclusion that the method or device is not safe. Therefore, we recommend, at least at the beginning, to use more the “minimum” button and with the generator at level 3 and, very important, to partially or fully relieve traction and tension in the moment of activation.

In confined spaces or near important structures (ILN, parathyroid, pharyngeal muscles, esophagus, jugular vein) the general rule of permanently seeing the tip of the instrument or, better, its whole active blade is crucial. The tip and the active blade of FCS9 become very hot if activated for more than 10 seconds. They can be cooled before the next usage by making contact with a wet gauze soaked in some cold sterile solution or even by directly immersing the instrument. When used to interrupt the Berry’s ligament, with the laryngeal entry point of the ILN clearly seen, a helpful artifice is to place a piece of wet sponge or gauze between the ligament and the nerve’s end.

Other “canons” to be strictly respected are: keep a safety margin of 5 mm., keep the active blade afar, use short activation times (less than 10 seconds) and a low power setting. FCS9 has been proven safe if all these precautions were respected [**15**, **19**].

In difficult cases, when the access to the superior vessels is very challenging, the surgeon is entitled to try an en masse ligation very close to the upper contour of the lobe. This is a situation when the usefulness of the FCS9 can be fully appreciated, but this altogether cutting must not become a rule. The safety dictum remains to treat the vessels separately, at their distal branches, on the surface of the superior pole, in order to preserve the integrity of the external branch of the superior laryngeal nerve.

**Fig. 2:**
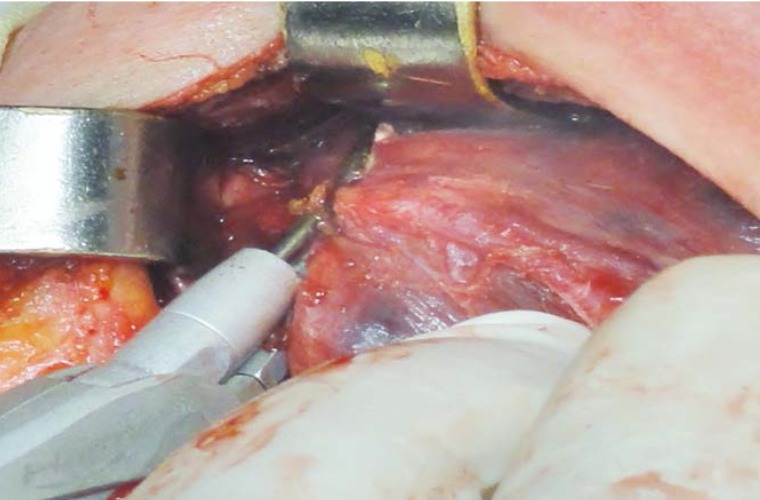
Dissection and division of the superior pedicle

**Fig. 3:**
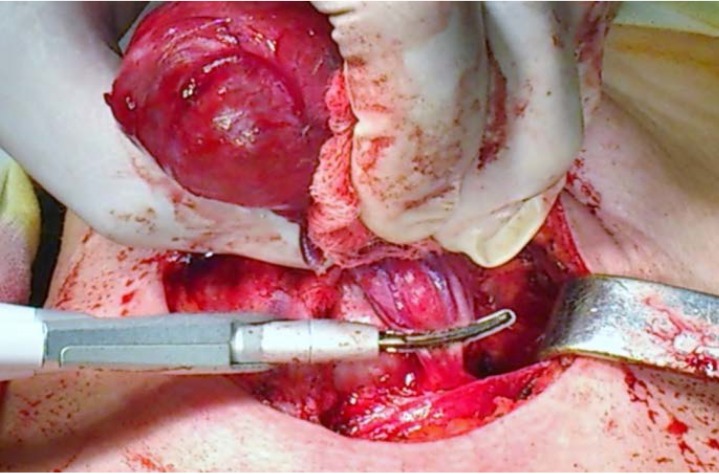
Dissection and division of the superior vessels

**Fig. 4:**
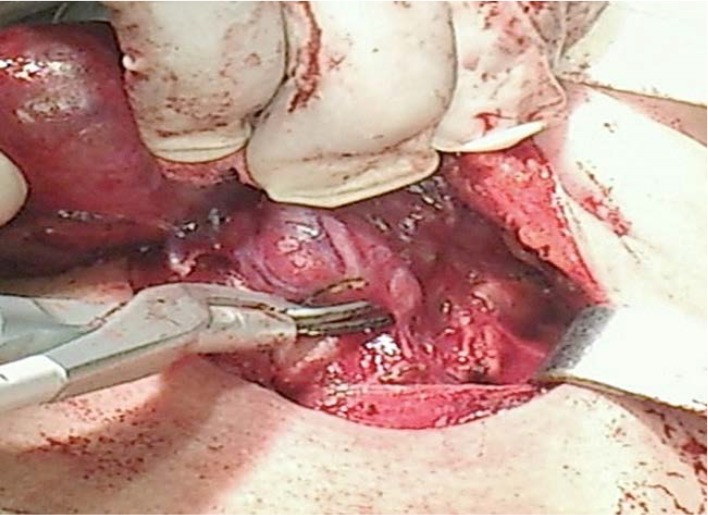
Dissection of the superior parathyroid gland

**Fig. 5:**
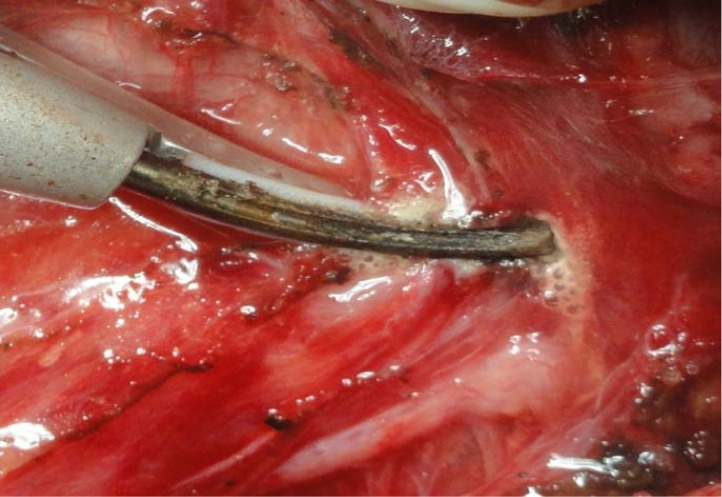
Division of the Berry’s ligament; ILN in close vicinity; minimal lateral thermal spread

**Fig. 6:**
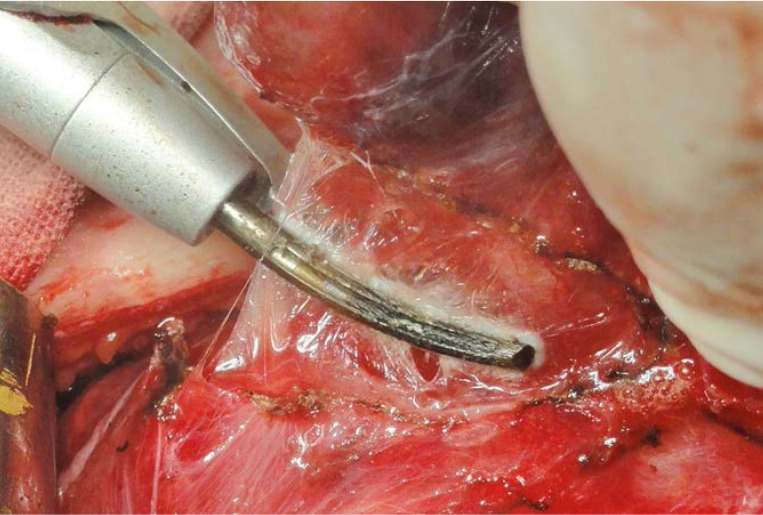
Separation of the gland from the trachea; minimal lateral thermal spread

Our surgical team gradually assimilated and respects all these rules and now uses the different hemostatic systems selectively. When deemed indicated, we employ FCS9 in all the stages of the procedure, including the dividing of the median raphe and strap muscles, of all the vessels, the perithyroideal dissection and mobilization of the gland, the exposing of the ILN, the dividing of the isthmus, the separation of the gland from the trachea (**Figures 2**, **3**, **4**, **5**, **6**).

The postoperative outcomes we have recorded are in reasonable limits, compared with data from the surgical literature (**Table 4**). Perhaps an important role had the rather large experience we have accumulated with the use of LigasurePrecise LS1200, more than 200 interventions.

**Table 4:** Postoperative outcomes compared with those from literature [**20**].

Complication	No. cases (incidence %) for our group of 50 patients	Incidence in surgical literature (reference no.20)
Intraoperative incidental bleeding	4 (8%)	NA
Postoperative hematoma – reoperation	1 (2%)	0.9 – 2%
Temporary symptomatic hypocalcemia	8 (17.4% of 46 total thyroidectomies)	16.4 – 29.6%
ILN paresis	4% (2 of 50 patients)	2.5 – 4.3%
	2% (2 of 96 nerves at risk)	3%

## Conclusion

After our initial experience with the use of HFCS9, we think we can conclude that it is a reliable instrument for obtaining an efficient, quick and safe hemostasis. As many studies have shown, the operative time is consistently reduced and we confirm that the fluency of the operation and comfort of the operating team are greatly enhanced. Its remarkable versatility makes it useable in all the steps of thyroidectomy. All these and some more underlined above, in such condition that the specific complications remain at low rates. However, what we think that has to be emphasized is that under no circumstances can this device (or any other device) be a substitute for an irreproachable and standardized technique and for a flawless knowledge of and respect for local anatomy. About gimmicks and gadgets in surgery it is worth recalling the words of a renown English surgeon, Sir Astley Paston Cooper, 1st Baronet (1768 – 1841): “If you are too fond of new remedies, first you will not cure your patients; secondly, you will have no patients to cure” (2). Moreover, after all, “a fool with a tool is still a fool” (2), meaning that the device can easily do harm if mishandled, that experience and training are essential.

**The authors have no conflicts of interest to disclose.**
